# β-Sitosterol Reduces the Content of Triglyceride and Cholesterol in a High-Fat Diet-Induced Non-Alcoholic Fatty Liver Disease Zebrafish (*Danio rerio*) Model

**DOI:** 10.3390/ani14091289

**Published:** 2024-04-25

**Authors:** Peng Zhang, Naicheng Liu, Mingyang Xue, Mengjie Zhang, Zidong Xiao, Chen Xu, Yuding Fan, Junqiang Qiu, Qinghua Zhang, Yong Zhou

**Affiliations:** 1 Yangtze River Fisheries Research Institute, Chinese Academy of Fishery Sciences, Wuhan 430223, China; somnium_zp@163.com (P.Z.); m200110460@st.shou.edu.cn (N.L.); xmy@yfi.ac.cn (M.X.); m200100231@st.shou.edu.cn (M.Z.); xiaohzau@163.com (Z.X.); xuchen@yfi.ac.cn (C.X.); fanyd@yfi.ac.cn (Y.F.); 2 Key Laboratory of Exploration and Utilization of Aquatic Genetic Resources, Ministry of Education, Shanghai Ocean University, Shanghai 201306, China; jqqiu@shou.edu.cn; 3 National Pathogen Collection Center for Aquatic Animals, Shanghai Ocean University, Shanghai 201306, China

**Keywords:** β-sitosterol, non-alcoholic fatty liver disease model, zebrafish, high-fat diet, blood fat untargeted lipidomics, lipid metabolism

## Abstract

**Simple Summary:**

β-sitosterol is a natural product with significant lipid-lowering and cholesterol-lowering effects. However, the mechanism of its action on aquatic products is not fully understood. We selected zebrafish as the research object. Through the observation of lipids in zebrafish, we found that β-sitosterol can reduce the accumulation of triglycerides and cholesterol in zebrafish, and reduce the related phenotypic changes caused by high-sugar and high-fat diet, thereby reducing lipid accumulation in zebrafish. This will provide a research basis for the development and use of β-sitosterol.

**Abstract:**

Objective: Non-alcoholic fatty liver disease (NAFLD) is strongly associated with hyperlipidemia, which is closely related to high levels of sugar and fat. β-sitosterol is a natural product with significant hypolipidemic and cholesterol-lowering effects. However, the underlying mechanism of its action on aquatic products is not completely understood. Methods: A high-fat diet (HFD)-induced NAFLD zebrafish model was successfully established, and the anti-hyperlipidemic effect and potential mechanism of β-sitosterol were studied using oil red O staining, filipin staining, and lipid metabolomics. Results: β-sitosterol significantly reduced the accumulation of triglyceride, glucose, and cholesterol in the zebrafish model. Kyoto Encyclopedia of Genes and Genomes (KEGG) pathway analysis showed that differential lipid molecules in β-sitosterol mainly regulated the lipid metabolism and signal transduction function of the zebrafish model. β-sitosterol mainly affected steroid biosynthesis and steroid hormone biosynthesis in the zebrafish model. Compared with the HFD group, the addition of 500 mg/100 g of β-sitosterol significantly inhibited the expression of *Ppar-γ* and *Rxr-α* in the zebrafish model by at least 50% and 25%, respectively. Conclusions: β-sitosterol can reduce lipid accumulation in the zebrafish model of NAFLD by regulating lipid metabolism and signal transduction and inhibiting adipogenesis and lipid storage.

## 1. Introduction

Lipids are essential nutrients for animal growth and play a vital role in the metabolism and immunity of organisms [1]. Lipids are considered essential nutrients for aquatic animals, providing fish with the energy needed for survival, and assume a significant role in aquatic nutrition [2]. The inclusion of lipids in aquatic feed has the potential to supply essential nutrients for optimal fish growth [3]. However, insufficient dietary lipid levels lead to the consumption of protein in feed for energy supply, resulting in decreased protein utilization. Conversely, a higher lipid content can effectively conserve protein and reduce production costs [4]. As a means of enhancing feed efficiency and minimizing protein consumption, the utilization of high-fat feed has become prevalent in aquaculture. Nevertheless, the adoption of a high-fat diet (HFD) may result in certain adverse effects. Specifically, the consumption of an HFD may result in certain adverse consequences. For instance, HFD may cause the accumulation of fat in the liver and abdomen of cultured fish, thereby inducing metabolic disorders [5,6]. Excessive fat intake can lead to weakened immunity, weakened disease resistance, and inflammatory responses in fish [5,7]. Additionally, it can induce lipid peroxidation in aquatic organisms and generate reactive oxygen species (ROS) [3]. Research has indicated that the provision of HFD may impede the growth performance and liver antioxidant capacity of largemouth bass (*Micropterus salmoides*), as well as diminish their immune response [8,9]. Similar studies in Atlantic salmon and rainbow trout have shown that higher fat intake reduces their growth performance [10,11,12]. Although studies have shown that a high-quality diet can promote the growth of fish, this is mainly due to increased fat deposition [13]. Therefore, the identification of appropriate supplements to mitigate the adverse consequences of a high-fat regimen is of paramount significance in the field of aquaculture.

β-sitosterol exhibits diverse physiological activities such as anti-inflammatory and antioxidant effects [14], hypolipidemic properties [15], sterol-lowering effects [16], etc. Research has demonstrated that β-sitosterol possesses hepatoprotective properties in mice, as evidenced by its ability to decrease cholesterol and triglyceride levels in a mouse model of HFD-induced nonalcoholic fatty liver disease (NAFLD) [17,18]. In the context of the rat model of type 2 diabetes mellitus (T2DM) induced by an HFD and streptozocin, the administration of β-sitosterol has been shown to stabilize blood glucose levels and reduce hyperglycemia. These findings suggest that β-sitosterol exhibits an insulin-like biological activity [15].

The accumulation of lipid metabolites is closely associated with obesity or obesity-related NAFLD [19]. NAFLD is often associated with metabolic syndrome features, such as dyslipidemia, hypertension, and T2DM [20]. The intrahepatic triglyceride accumulation (i.e., steatosis, the hallmark feature of NAFLD) results from an imbalance between complex molecular pathways of lipid metabolism [20]. Animal models are critical tools for studying NAFLD, as well as the development of therapeutic drugs and prevention and treatment strategies [20]. There are several studies on NAFLD model organisms, such as rats [15], mice [21], and zebrafish [22]. Zebrafish are relatively inexpensive and easy to use and maintain compared with mammalian models [23]. They develop quickly and are opticrpmally transparent, allowing for easy observations of phenotypic responses [23]. An HFD-induced obese zebrafish model exhibited clinical manifestations of NAFLD, including hyperinsulinemia and impaired glucose tolerance [24]. The lipid metabolism process in zebrafish, encompassing lipid absorption and transport, oxidative metabolism, and related processes, bears a striking resemblance to that of mammals [25], and it can also reveal the changes in lipid metabolism in fish. These benefits render zebrafish highly suitable models for investigating diseases associated with lipid metabolism, such as diabetes and NAFLD [26,27]. NAFLD is a metabolic disorder with multiple causes, long-term hyperglycemia causes macrovascular or microvascular complications, such as retinopathy [28]. Meanwhile, a zebrafish model of HFD-induced NAFLD has achieved good application progress [20]. Therefore, in a diet-induced zebrafish model of NAFLD, the establishment of the model can be judged by examining the changes in body indicators, such as lipid changes [24], genetic changes [29], retinal vascular changes [28], etc. Moreover, zebrafish lipidomics has been extensively employed in diverse fields such as drug screening, metabolic syndrome, etc. [30,31,32]. The present study constructed an NAFLD model by administering HFD to both zebrafish larvae and adults. The impact of β-sitosterol on lipid metabolism in aquatic organisms was then investigated using lipidomic analysis. The findings of this study offer empirical evidence to support the use of β-sitosterol as a feed supplement to enhance lipid metabolism in cultured fish.

## 2. Materials and Methods

### 2.1. Reagents

β-sitosterol was purchased from Shanghai YuanYe Biotechnology Co., Ltd. (Shanghai, China). Oil red O stain solution, filipin stain solution, dimethyl sulfoxide (DMSO), tricaine methanesulfonate (MS222), and phenylthiourea (PTU) were purchased from Sigma-Aldrich (St. Louis, MO, USA). Cholesterol, egg yolk powder, and glucose were purchased from Sangon (Shanghai, China). Zebrafish feed was purchased from Shengsuo Co., Ltd. (Yantai, Shandong, China).

### 2.2. Zebrafish Stocks and Rearing Conditions

AB and *Tg* (*fli1a*: *EGFP*) zebrafish strains were procured from the National Zebrafish Resource Center, which is affiliated with the Institute of Aquatic Biology at the Chinese Academy of Sciences (Wuhan, China). Zebrafish were maintained according to standard protocols (zfin.org). Adult and larval zebrafish were fed regularly twice a day. Before mating, an equal number of male and female zebrafish was introduced into a designated mating tank. The subsequent day, natural spawning occurred, and the resulting embryos were collected. Following microscopic examination, fertilized eggs were chosen and subsequently incubated at 28 °C, with the E3 culture medium (Nanjing EzeRinka Biotechnology, Nanjing, China) replaced every 24 h. All animal experiments were approved by the Animal Experimental Ethical Inspection of Laboratory Animal Centre, Yangtze River Fisheries Research Institute, Chinese Academy of Fishery Sciences (ID number: YFI 2022-zhouyong-1201).

### 2.3. Preparation of Reagents

To prepare a stock solution of 1 mg/mL, β-sitosterol was dissolved in a mixture of DMSO (30%) and E3 culture medium (70%). The resulting solution was then utilized to create a working solution of the desired concentration. All the remaining reagents were prepared according to the instructions.

### 2.4. Preparation of HFD and Establishment of a Zebrafish Model of NAFLD

Formulation of larval zebrafish HFD: Following the dissolution of 10 g of egg yolk powder and 1 g of cholesterol, the resulting mixture was homogenized on a magnetic stirrer and subsequently dried overnight in a freeze dryer (SCIENTE, Ningbo, China).

Formulation of adult zebrafish HFD: After the dissolution of 10 g of egg yolk powder, 10 g of cholesterol, and 3 g of glucose, the resulting mixture was homogenized with 100 g of zebrafish feed using a magnetic stirrer. Subsequently, the mixture was dried overnight in a freeze-dryer (SCIENTE, Ningbo, China). The HFD for the experimental group was enriched with 50 mg of β-sitosterol, while the control group received an equivalent amount of DMSO as the β-sitosterol group.

Construction of a larval zebrafish model of NAFLD: Fish were raised to 5 days post-fertilization (dpf) in an E3 medium, with 0.003% 1-phenyl-2-thiourea (PTU) added at 22 h post-fertilization (hpf) to clear pigment [33]. Zebrafish at 5 dpf were selected for the experiment, and the larvae were fed with HFD twice a day for 5 days. During feeding, 3% glucose solution was added for soaking, and the feeding time was 1 h. After feeding, a fresh E3 culture medium was replaced. The experimental group was given 200 μg/mL of β-sitosterol, the positive control group received 62.5 μg/mL of bezafibrate (a peroxisome proliferator-activated receptor activator that reduces blood lipids), and the control group received the same amount of DMSO as the β-sitosterol group. Subsequently, the accumulation of triglyceride and cholesterol in zebrafish larvae was detected [29]. Toxicity tests were used to determine bezafibrate and β-sitosterol concentrations.

Construction of an adult zebrafish model of NAFLD: This model was established by a feeding regimen of HFD administered twice daily for one month. Subsequently, the liver of the zebrafish was collected to determine the levels of cholesterol and triglyceride, followed by statistical analysis to confirm the establishment of the model [34].

### 2.5. Oil Red O Staining of Zebrafish Larvae

Oil red O staining was utilized to detect the accumulation of triglycerides [35]. Zebrafish were euthanized using MS-222 (4%) [36] and subsequently fixed with Bouin’s solution (Sigma, St. Louis, MO, USA). The zebrafish were then fixed at 4 °C for 24 h, followed by washing with 1× phosphate-buffered saline with Tween (PBST) for five cycles, each lasting 15 min. The samples were dehydrated using a series of methanol solutions: 25% methanol (containing 75% PBST), 50% methanol (containing 50% PBST), 75% methanol (containing 25% PBST), and 100% methanol, each for 15 min. Subsequently, the zebrafish were immersed in a solution of oil red dye (0.5% methanol) overnight, followed by incubation in methanol solutions of varying concentrations: 100% and 75% (containing 25% PBST), 50% (containing 50% PBST), and 25% methanol. Samples were photographed under a microscope (Olympus, Tokyo, Japan). Image J 1.48 software was utilized to process the image and determine the grayscale. Subsequently, a difference analysis was conducted. Six zebrafish were used in each group.

### 2.6. Filipin Staining of Zebrafish Larvae

The accumulation of triglycerides was detected by filipin staining [37]. Zebrafish were anesthetized using MS-222 (4%) [36] and subsequently fixed with Bouin’s solution. The fixation process was carried out in a refrigerator at 4 °C for 24 h. Following fixation, the specimens were washed five times in 1 × PBST, for 15 min each time. A working solution of filipin at a concentration of 50 μg/mL was added to the specimens and allowed to react in the dark for 30 min. The specimens were washed five times in 1 × PBST and then photographed under a microscope. The fluorescence intensity was calculated from the processed fluorescence images using Image J 1.48 software. Afterward, the difference analysis was carried out. Six zebrafish were used in each group.

### 2.7. Zebrafish Eyeball Lens Extraction and Vascular Diameter Statistics

The blood vessel diameter of each lens was measured in the zebrafish [28]. Cell samples from the modeled zebrafish larvae were fixed in 4% paraformaldehyde at 4 °C for 24 h. Subsequently, they were washed thrice with distilled water, for 20 min each time. Cells were then incubated with 3% trypsin (Tris-HCl, pH 7.8) at 37 °C for 80 min, with gentle reversal every 20 min. The digestion process was terminated, and the lens was dissected under a dissecting microscope (Olympus, Tokyo, Japan). The vascular images of the lens were scanned using single-photon laser confocal microscopy (Olympus, Tokyo, Japan). The diameter of the vitreous vessels was calculated using Image J 1.48 software. Subsequently, a difference analysis was conducted. Six zebrafish were used in each group.

### 2.8. Quantification of Cholesterol, Glucose, and Triglyceride Levels in Hepatic Tissue of Adult Zebrafish

After euthanizing zebrafish with MS-222, the liver tissue of zebrafish was extracted and subsequently diluted with sterile phosphate-buffered saline to obtain a tissue homogenate concentration of 1%. The resultant mixture was subjected to centrifugation at 5000× *g* at 4 °C for 20 min, yielding a supernatant that was utilized for subsequent analysis [38]. Protein concentrations in the tissue homogenate were measured using a bicinchoninic acid (BCA) kit (Biyuntian, Shanghai, China). Subsequently, cholesterol and triglyceride levels in the tissue homogenate were evaluated using tissue triglyceride and cholesterol assay kits (Nanjing Jiancheng Bioengineering Institute, Nanjing, China). The data were analyzed for significant differences. Each group consisted of three biological replicates, with 30 zebrafish in each group.

### 2.9. Enzyme-Linked Immunosorbent Assay (ELISA)

The liver of zebrafish was subjected to ELISA to quantify the expression of *Ppar-γ* and *Rxr-α*. Each group consisted of three biological replicates, with 30 zebrafish in each group, which were homogenized in cold phosphate buffer (with a ratio of 1 g tissue sample to 9 mL of phosphate buffer) at pH 7.4. After centrifugation at 3000× *g* at 4 °C for 10 min, the supernatant was collected, and the contents of *Ppar-γ* and *Rxr-α* were analyzed using corresponding ELISA kits (Jianglai biology, Shanghai, China) following the manufacturer’s instructions.

### 2.10. Lipidomics Sample Preparation and Analysis

Adult zebrafish in the blank control group (BC), HFD group (HFD), and β-sitosterol group (B) were euthanized after an ice water bath [39]. Subsequently, the liver tissue was obtained and transferred to an enzyme-free EP tube and cryopreserved in liquid nitrogen. Each group consisted of three biological replicates, with nine liver tissue samples in each replicate. One portion of the tissue samples was sent to Biomarker Technologies (Shandong, China) for lipidomic sequencing and data analysis, whereas the other portion was used for subsequent analysis.

### 2.11. Reverse Transcription-Quantitative Polymerase Chain Reaction (RT-qPCR)

Total RNA was extracted using TRIzol reagent [40]. RNA integrity was determined by electrophoresis using 1.5% agarose gels. The concentration (A260) and purity (A260/A280, A260/A230) of the RNAs were measured using a NanoDrop 1000 instrument (Thermo Fisher Scientific, Wilmington, DE, USA). The RNA was reverse transcribed into complementary DNA (cDNA) using a cDNA reverse transcription kit (Trans Gen Biotech, Shanghai, China). Each group consisted of three biological replicates, with three liver tissue samples in each replicate. RT-qPCR was conducted under standard cycle conditions, consisting of an initial denaturation step at 95 °C for 10 min, followed by 40 cycles of amplification at 95 °C for 30 s and annealing/extension at 60 °C for 30 s. The 2^−ΔΔCt^ method was employed for data analysis [41]. Primer sequences used in this study are shown in Table 1. *β*-*actin* is the housekeeping gene used in this study.

### 2.12. Statistical Analysis

Differences between groups were compared using a *t*-test or one-way analysis of variance (ANOVA) and the least significant difference test. A probability level of 5% (*p* < 0.05) was deemed significant. In instances where the normality test was not met, Kruskal–Wallis non-parametric one-way ANOVA was utilized, and differences between groups were assessed using the Mann–Whitney test.

## 3. Results

### 3.1. β-Sitosterol Reduces Triglyceride and Cholesterol Levels in Zebrafish

**Figure 1:**
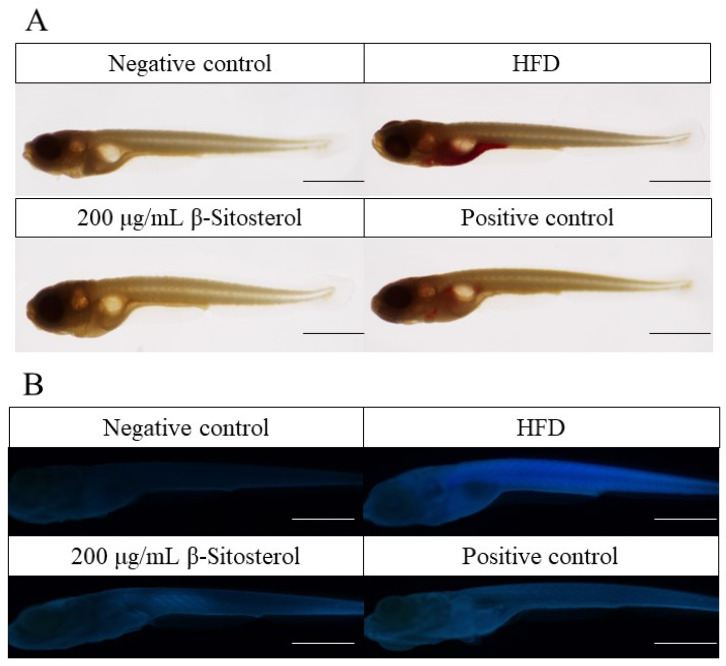

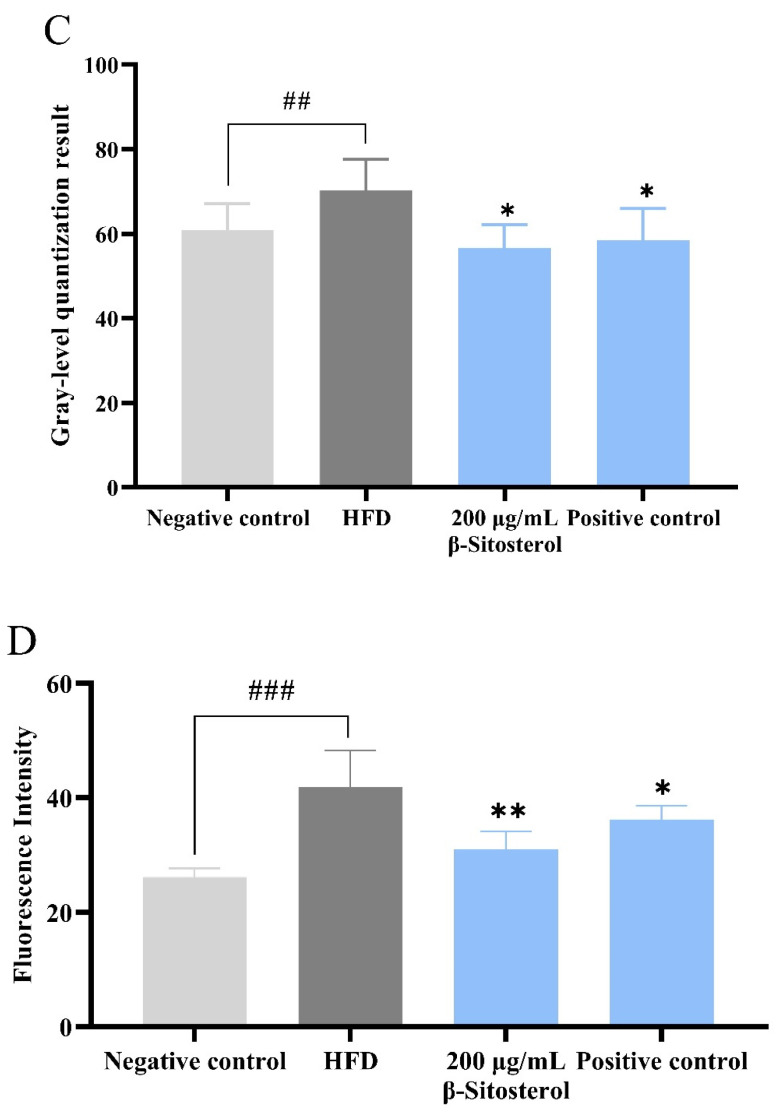
β-sitosterol reduces the content of triglyceride and cholesterol in zebrafish. (**A**) Oil red O staining results, scale: 1 mm. (**B**) Filipin staining results, scale: 1 mm. (**C**) Quantitative results of oil red O staining, *n* = 6. (**D**) Quantitative results of Filipin staining, *n* = 6. Results are expressed as mean ± SE of the three repeated samples. ^##^ *p* < 0.05, ^###^ *p* < 0.01 compared with the negative control group; * *p* < 0.05, ** *p* < 0.01 compared with the HFD group.

Triglycerides were predominantly stored in the visceral, intestinal, and aortic regions of the heart in the HFD group. Oil red O and filipin staining results showed that the fluorescence intensity of cholesterol was markedly elevated in the HFD group compared with the negative control group (Figure 1A,B). The accumulation of triglycerides and cholesterol in the abdominal region was considerably mitigated after the administration of the positive drug bezafibrate, although a residual amount of triglyceride deposition persisted in the localized area (Figure 1A,B). Similarly, β-sitosterol administration did not result in significant triglyceride accumulation in the abdominal region (Figure 1A). The fluorescence intensity of cholesterol staining exhibited a significant reduction (Figure 1B). Simultaneously, combined with quantitative analysis results, it was discovered that β-sitosterol significantly decreased triglyceride and cholesterol levels in zebrafish (Figure 1C,D).

### 3.2. Effects of β-Sitosterol on the Microvessels of the Zebrafish Vitreous Vascular System

**Figure 2:**
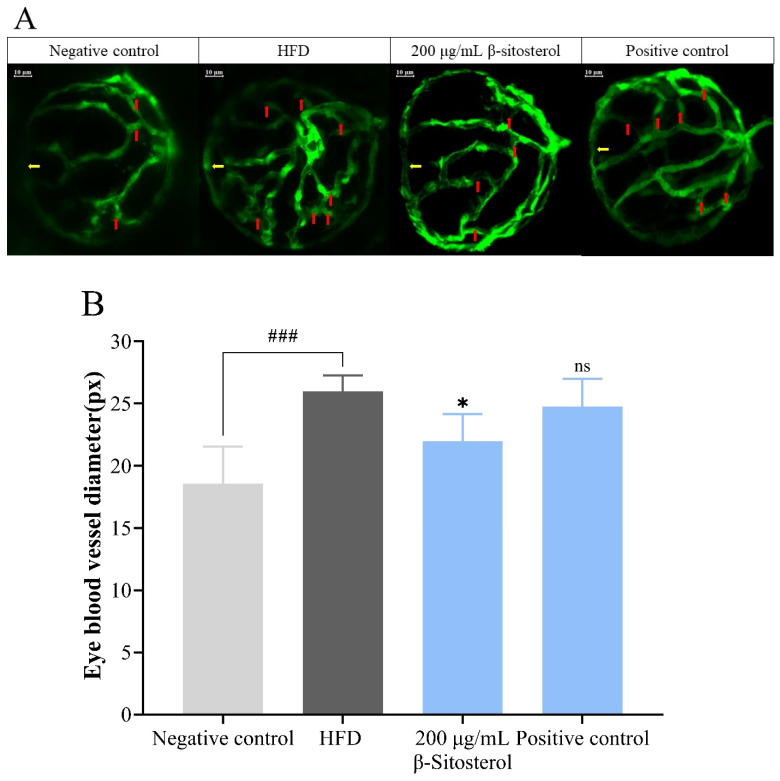
Changes in microvessels in the zebrafish vitreous vascular system. (**A**) Confocal scan of the outer vasculature surrounding the ocular globe in *Tg* (*fli1a*: *EGFP*) zebrafish larvae. Through the translucent lens, transparent blood vessels (yellow arrows in (**A**)) can be seen from the outside, and red arrows indicate the branches between the vascular arcs. Scale: 10 μm. (**B**) Quantitative analysis of the vascular diameter in confocal scanning images, *n* = 6. The unit of length is in pixels (px). ^###^ *p* < 0.01 compared with the negative control group; * *p* < 0.05 compared with HFD group; ns, no significant difference.

The lipid content alteration in zebrafish blood was assessed by measuring the blood vessel diameter of each lens [28]. As shown in Figure 2A, vascular branches were significantly fewer in the negative control group than in the HFD group. Meanwhile, bezafibrate administration significantly reduced the branch density, and similar results were observed after treatment with β-sitosterol (Figure 2A). The blood vessel diameter at the location indicated by the yellow arrow (Figure 2A) was reassessed for further analysis. The results showed that compared with the control group, the vascular diameter was significantly increased in the HFD group. However, β-sitosterol treatment significantly reduced the vascular diameter compared with the HFD group. No significant change was detected in the bezafibrate group, which may be attributed to the low concentration (Figure 2B).

### 3.3. Effects of β-Sitosterol on Triglyceride and Cholesterol Levels in Adult Zebrafish Liver

**Figure 3:**
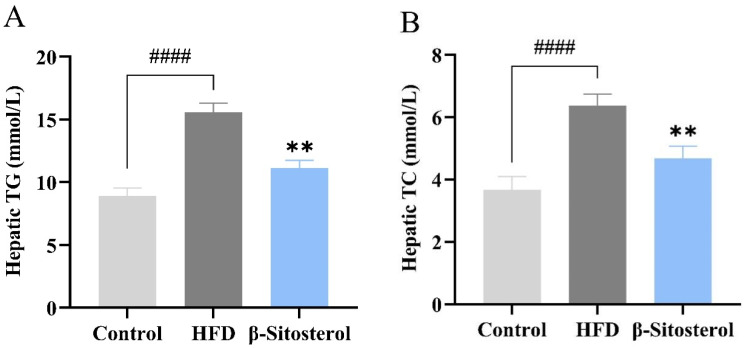
β-Sitosterol reduces the contents of triglyceride and cholesterol in the liver of zebrafish. (**A**) Triglyceride content in the liver of zebrafish. (**B**) Cholesterol content in the liver of adult zebrafish. Results are expressed as mean ± SE of the three repeated samples. ^####^ *p* < 0.01 compared with the control group; ** *p* < 0.01 compared with the HFD group.

Triglyceride and cholesterol levels in the liver of adult zebrafish were assessed using tissue triglyceride and cholesterol assay kits to evaluate the establishment of a zebrafish model of NAFLD. The results showed that the HFD group exhibited a significant increase in both triglyceride and cholesterol contents (Figure 3A,B). Specifically, the triglyceride content reached 15.6 mmol/L (Figure 3A) and the cholesterol content reached 6.3 mmol/L (Figure 3B). However, β-sitosterol treatment significantly reduced the contents of both triglyceride and cholesterol in the liver of adult zebrafish (Figure 3A,B). The triglyceride content was 11.1 mmol/L, and the cholesterol content was 4.7 mmol/L, which decreased by 29% and 25%, respectively. Overall, these findings indicate that β-sitosterol can decrease the levels of triglycerides and cholesterol in the liver of adult zebrafish. Furthermore, an adult zebrafish model of NAFLD was successfully established and used for subsequent lipid metabolism analysis.

### 3.4. Lipid Metabolome Analysis of Adult Zebrafish Liver

**Figure 4:**
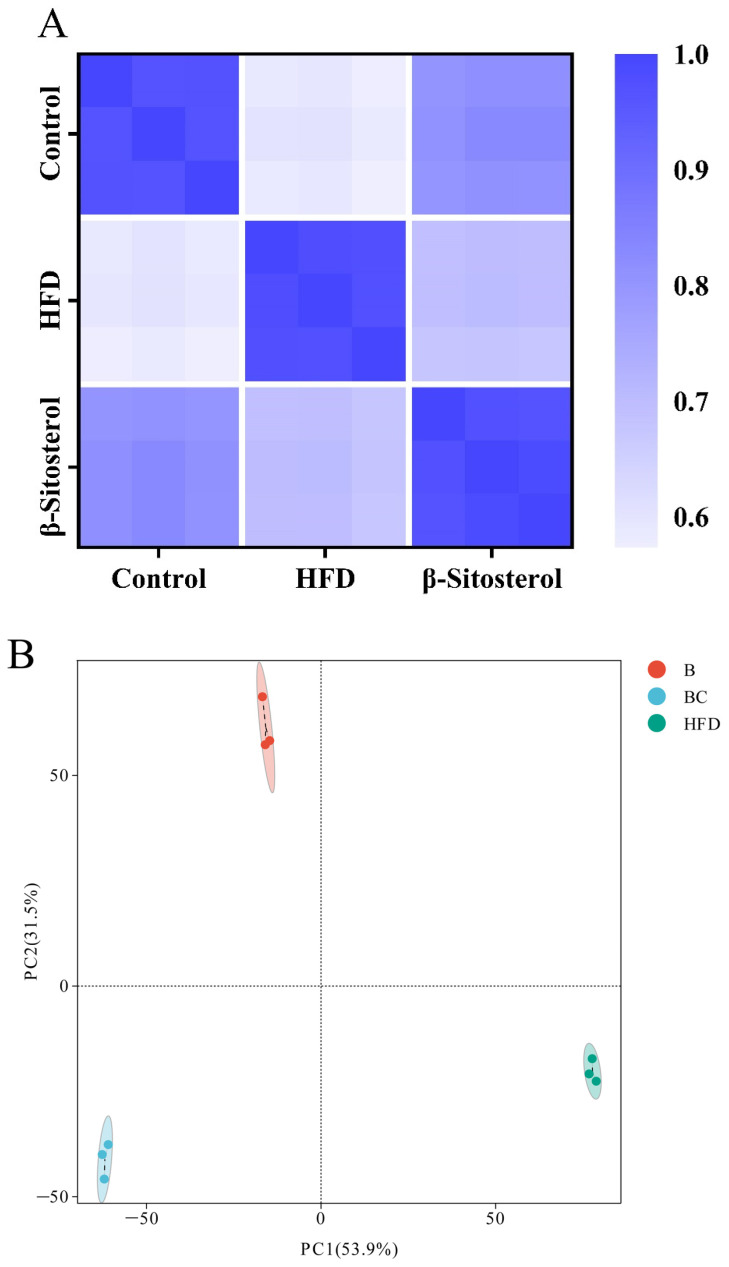

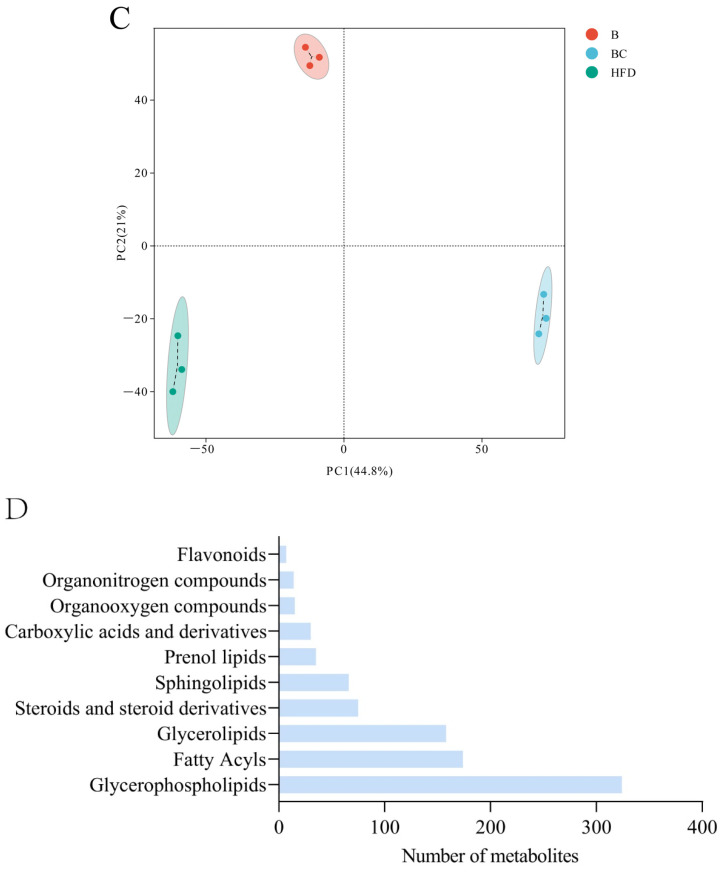
Lipid metabolome analysis of adult zebrafish liver. (**A**) Correlation analysis between samples in control, HFD, and β-sitosterol groups. (**B**) PCA analysis results in the positive POS ion mode. The *X*-axis represents the first principal component, and the *Y*-axis represents the second principal component. (**C**) PCA analysis results in NEG ion mode. (**D**) Lipid classification of the top 10 metabolites. B, β-sitosterol group; BC, blank control group; HFD, HFD group; PCA, principal component analysis; POS, positive; NEG, negative.

Correlation analysis was performed among the control, HFD, and β-sitosterol groups. The square of the Spearman rank correlation coefficient rho (r) served as the evaluation index for biological repeated correlation. As shown in Figure 4A, the r^2^ value of the samples within the group was proximate to 1, signifying a high degree of similarity among the samples within the group. Conversely, the r^2^ value between the samples across different groups was comparatively low, indicating a low level of similarity among the samples between the groups. This suggests that the sample’s reliability is exceedingly high.

Principal component analysis (PCA) was employed to assess the overall metabolic differences and degree of variation between samples within each group. The analysis was conducted separately in both positive (POS) and negative (NEG) ion modes. The results from the PCA showed complete segregation of control, HFD, and β-sitosterol groups, suggesting a significant alteration in metabolite levels among the three groups (Figure 4B,C). The classification results revealed that glycerophospholipids constituted the highest proportion of metabolites among the three groups, followed by fatty acyls and glycerolipids (Figure 4D). A detailed metabolite classification is presented in Supplementary Table S1.

### 3.5. Analysis of Differentially Expressed Lipid Metabolites (DELMs) in Zebrafish Liver of HFD and β-Sitosterol Groups

**Figure 5:**
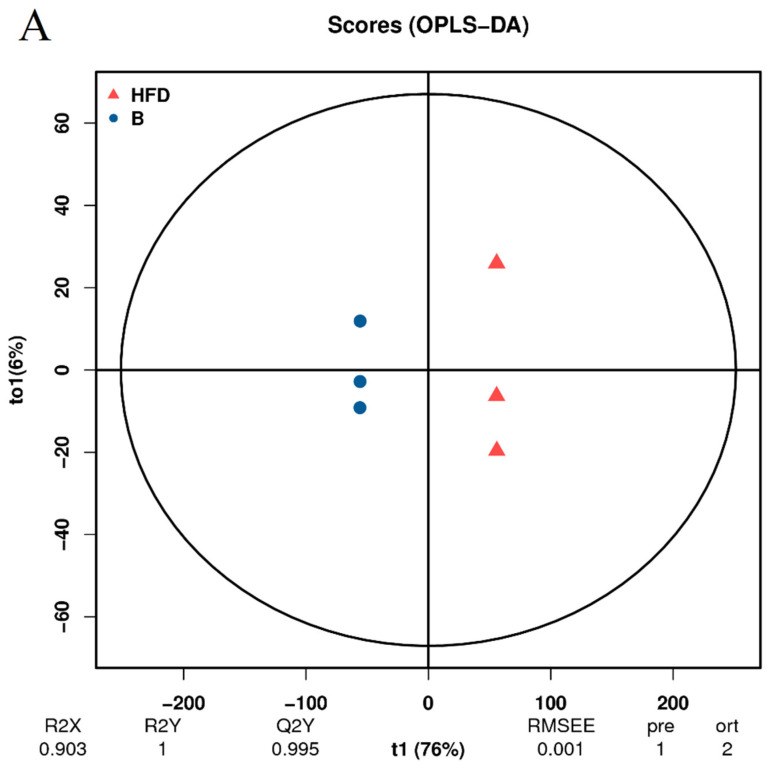

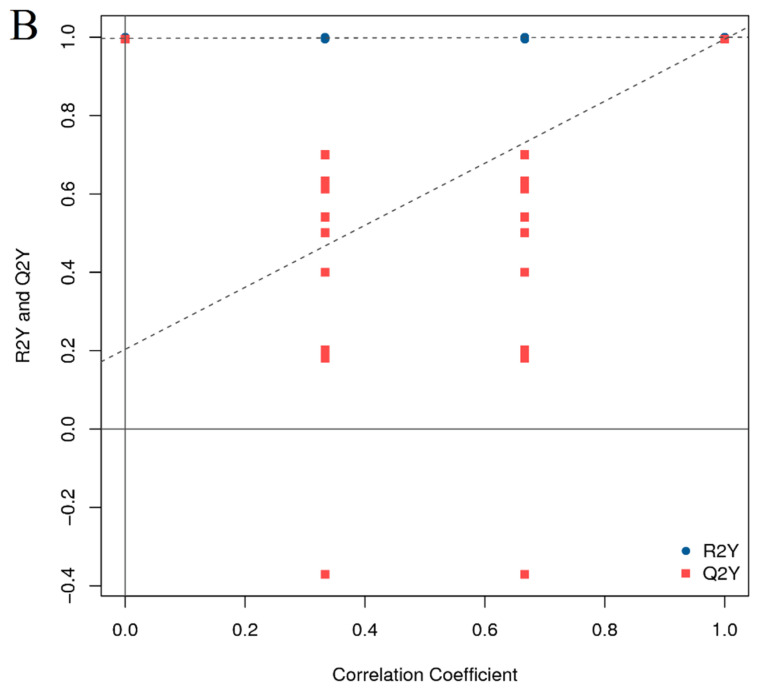
Orthogonal projections to latent structures discriminant analysis (OPLS-DA). (**A**) OPLS-DA score diagram. The *x*-axis (t1) represents the prediction component (inter-group difference component), and the *y*-axis (to1) represents the orthogonal component (intra-group difference component). (**B**) OPLS-DA model replacement test diagram. The *x*-axis represents the correlation between the permutation group and the original model group, the *y*-axis represents the value of R2Y or Q2Y (where R2Y and Q2Y of 1 in the *x*-axis are the values of the original model), and the two dashed lines are the regression lines fitted by R2Y and Q2Y.

The preceding analysis has demonstrated the successful establishment of the HFD group model (Figure 3A,B). To further investigate the impact of β-sitosterol on zebrafish with high-sugar and HFDs, two groups were selected for subsequent analysis: the HFD group and the β-sitosterol group. The orthogonal projections to latent structures discriminant analysis (OPLS-DA) methodology is similar to PCA analysis. OPLS-DA is suitable for diagnosing dissimilarities between sample groups based on the outcomes of mass spectrometry analysis and can ascertain anomalous experimental samples through the analysis of their dispersion patterns [42]. The evaluation model utilizes R2X, R2Y, and Q2Y as prediction parameters. R2X and R2Y indicate the model’s interpretation rate of the X and Y matrices, respectively. The X matrix serves as the model input, specifically the lipid quantitative matrix, while the Y matrix functions as the model output, specifically the sample grouping matrix. The predictive capacity of the model is denoted by Q2Y, which determines the model’s ability to accurately differentiate sample groupings based on metabolic expression. A higher value of R2Y and Q2Y in the index indicates greater stability and dependability of the model, with the ability to effectively screen DELMs. An exemplary model is denoted by Q2Y > 0.9. The results showed that the value of Q2Y and R2Y was 0.995 and 1, respectively (Figure 5A), signifying the stability and dependability of the experimental model. To ensure the reliability of the OPLS-DA model, a permutation test was conducted. The grouping of samples is randomly disrupted (replaced) and OPLS-DA modeling is executed based on the permutation group, with R2Y and Q2Y being computed. The results of multiple modeling are presented as scatter plots. As shown in Figure 5B, the Q2Y fitting regression line exhibited a positive slope and the R2Y point was typically positioned above the Q2Y point, suggesting that the model is both significant and autonomous. The results of PCA (Figure 4B,C) and OPLS-DA (Figure 5A,B) showed a complete separation between the HFD group and the β-sitosterol group, suggesting a significant alteration in the lipid metabolite levels between the two groups.

### 3.6. Analysis of DELMs in Zebrafish Liver of HFD and β-Sitosterol Groups

**Figure 6:**
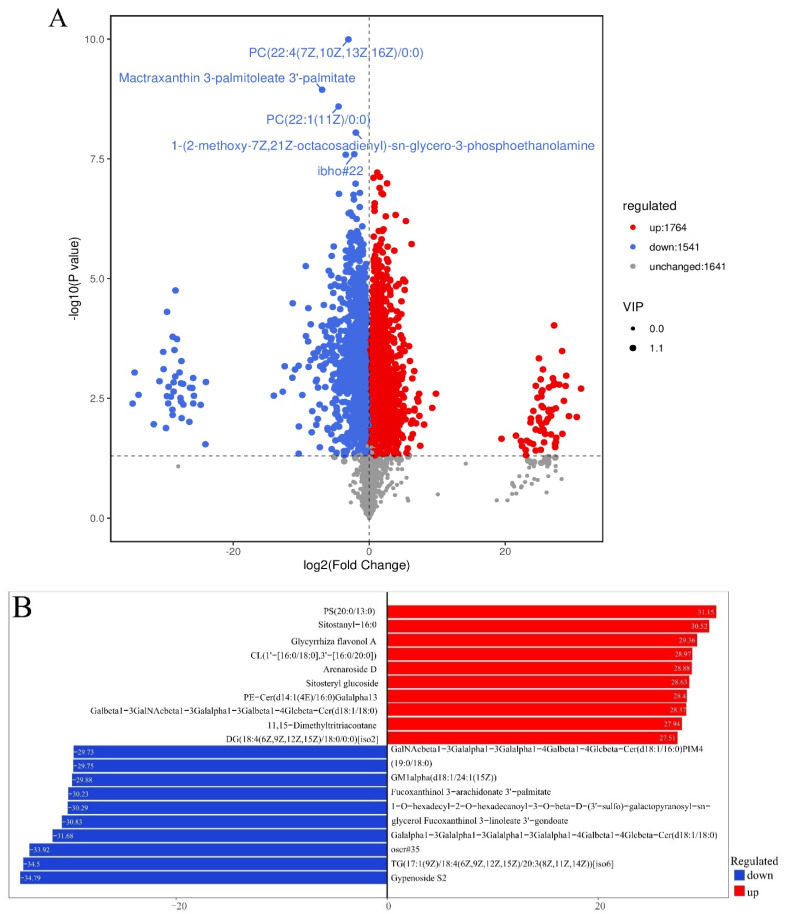
Analysis of differentially expressed lipid metabolites (DELMs) between HFD and β-sitosterol groups. (**A**) Volcano plot results; the *x*-axis represents the difference in multiple changes in the group compared with each substance (log_2_), and the *y*-axis represents the *p*-value (log_10_). The size of the scatter represents the VIP value of the OPLS-DA model. The larger the scatter point, the larger the VIP value, and the more reliable the DELMs screened. The blue dots in the figure represent downregulated DELMs, the red dots represent upregulated DELMs, and the gray represents the detected non-significant lipids. (**B**) Top 10 upregulated and downregulated lipid metabolites based on log_2_FC. The *x*-axis represents the change in the difference multiples of each substance in the group (taking log_2_ as the bottom) based on the upregulation and downregulation; upregulation is represented by the red color, downregulation is indicated by the blue color, and logFC is represented by the column length.

Based on the results of OPLS-DA, the variable importance in projection (VIP) values of OPLS-DA multivariate analysis were used to preliminarily screen out lipids with different varieties or tissues [43]. Meanwhile, the *p*-value or fold change (FC) obtained from univariate analysis was integrated to effectively identify DELMs. FC ≥ 1, VIP ≥ 1, and *p*-value < 0.05 were set as the screening criteria to screen DELMs [43]. A combination of POS and NEG ion modes yielded 3305 DELMs in the B group compared with the HFD group, of which 1764 metabolites were upregulated and 1541 were downregulated (Figure 6A). The volcano plot showed that the five metabolites exhibiting the most prominent changes were downregulated and classified as glycerophospholipids (GP), propanol lipids (PR), glycerophospholipids (GP), and fatty acyl (FA) (Figure 6A). A detailed metabolite classification is displayed in Supplementary Table S2. Preliminary assessment suggests that β-sitosterol exhibits the most prominent downregulation of glycerophospholipid metabolites. Figure 6B summarizes the lipid logFC results of the top 10 upregulated and downregulated lipid metabolites in the experimental group compared with the control group, obtained through differential metabolite analysis and log conversion processing of the difference multiples. The results primarily comprise GP, sterol lipids (ST), polyketides (PK), adrenergic lipids (PR), sphingolipids (SP), fatty acyl (FA), and glycerolipids (GL). A detailed metabolite classification is displayed in Supplementary Table S3.

### 3.7. KEGG Functional Annotation and Enrichment Analysis of DELMs

**Figure 7:**
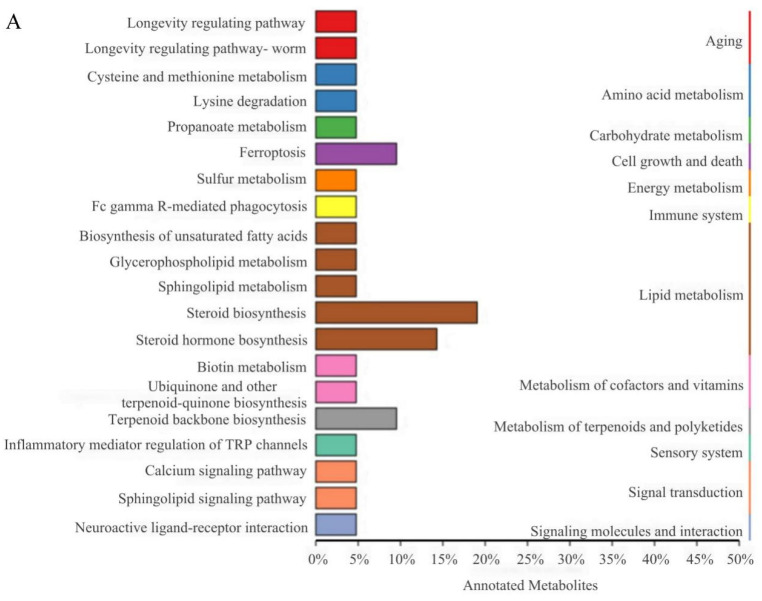

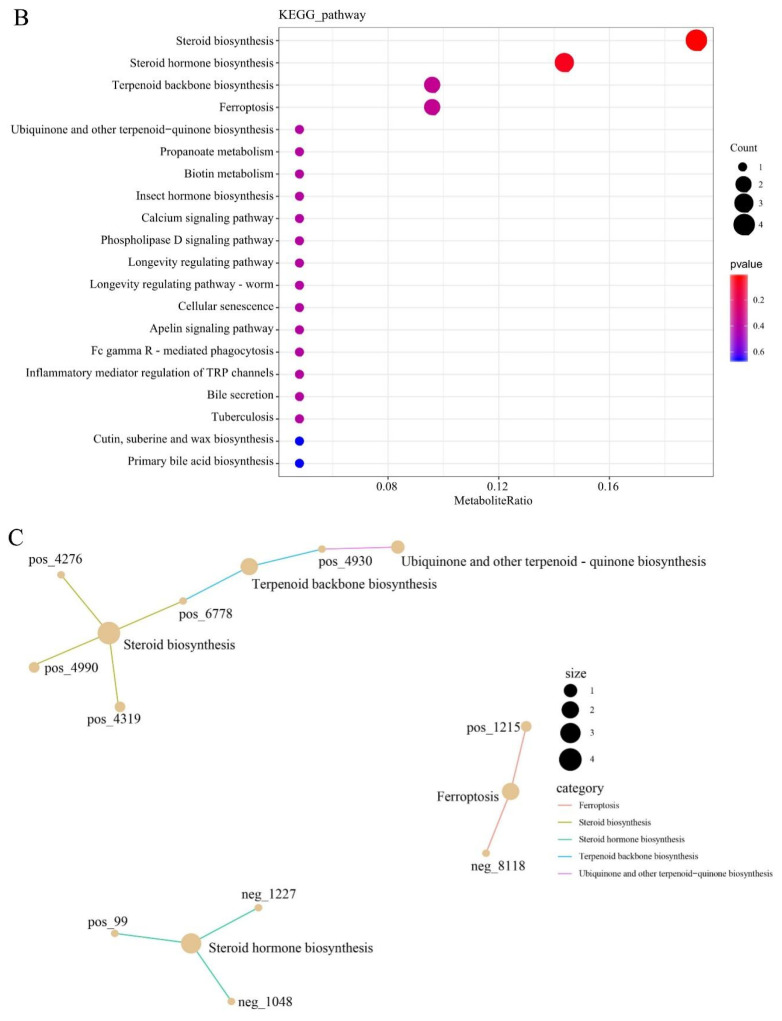

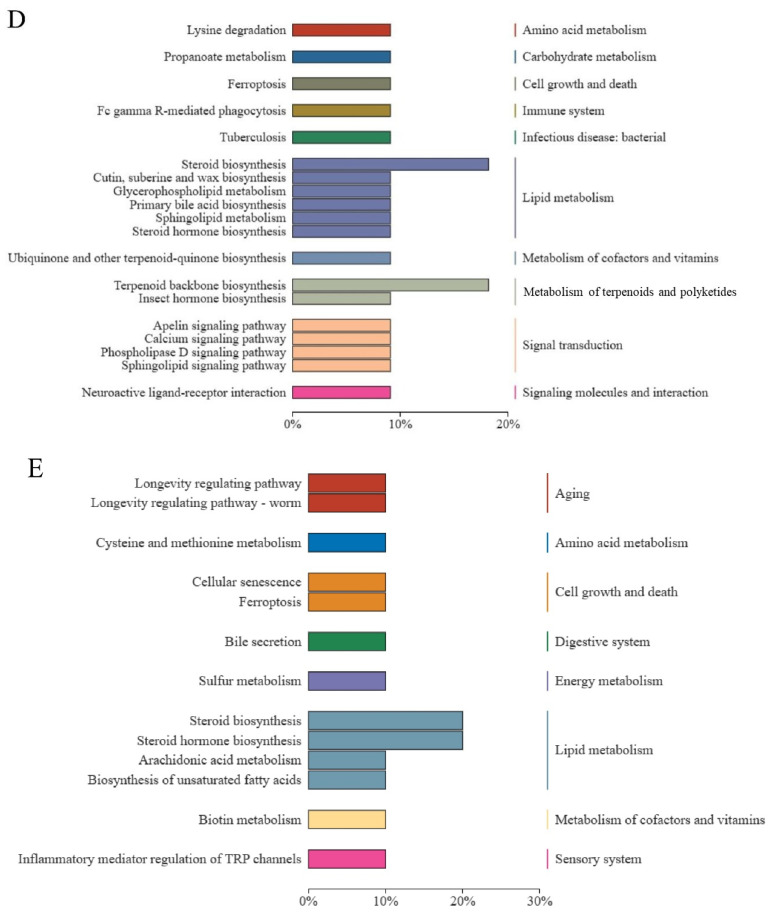
Differential lipid KEGG functional annotation and enrichment analysis results. (**A**) The annotation results of the TOP20 differential lipid KEGG enrichment. The entries shown in the same box indicate the hierarchical classification annotation of the KEGG pathway, and the column length shows the number of lipids annotated to each pathway. (**B**) Differential lipid KEGG annotation results for the TOP20 enrichment point map. (**C**) Differential lipid KEGG annotation results from TOP20 enrichment network map. (**D**) KEGG functional annotation and enrichment analysis results of the upregulated metabolites in differential lipids. (**E**) KEGG functional annotation and enrichment analysis results of the downregulated metabolites in differential lipids.

KEGG pathway and pathway-based network analyses of DELMs were performed. DELMs were annotated using the KEGG database, and the top 20 most significant DELMs within the pathway were selected. The enrichment of DELMs was primarily observed in lipid metabolism (Figure 7A), as well as in steroid biosynthesis and steroid hormone biosynthesis (Figure 7B). The enrichment network diagram revealed that the processes driving the synthesis of steroids, terpenoid backbone, ubiquinone, and other terpenoid–quinone are intricately interconnected (Figure 7C). This observation may be attributed to the potential influence of certain differential metabolites on multiple metabolic pathways simultaneously. Figure 7D shows the enhanced biological functions of the B group compared with the HFD group. Notably, upregulated metabolites were primarily involved in lipid metabolism and signal transduction (Figure 7D). Moreover, downregulated metabolites were primarily involved in lipid metabolism of the biological functions (Figure 7E). Since the function of enrichment is not only a single metabolite, there are upregulation and downregulation of steroid biosynthesis and steroid hormone biosynthesis in lipid metabolism. Collectively, these data suggest that β-sitosterol can affect lipid metabolism and other pathways in zebrafish, particularly those related to steroid and steroid hormone biosynthesis.

### 3.8. Effects of β-Sitosterol Treatment on Ppar-γ and Rxr-α in Adult Zebrafish Liver

**Figure 8:**
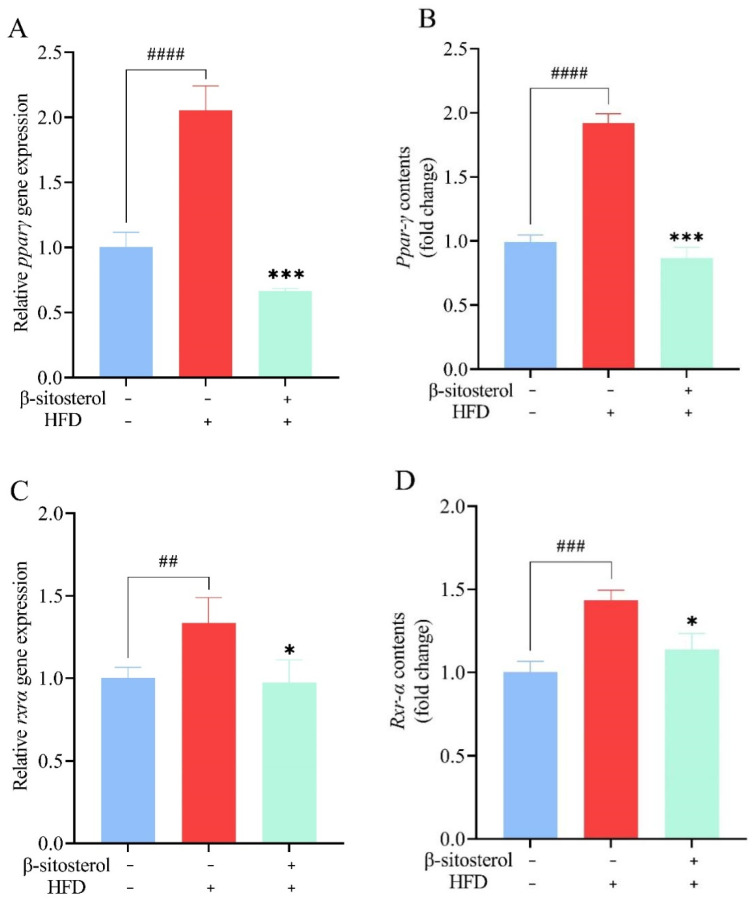
Effects of β-sitosterol on the expression of *Ppar-γ* and *Rxr-α* in zebrafish. (**A**) The mRNA level of *ppar-γ* in the liver. (**B**) The mRNA level of *Ppar-γ* in the liver. (**C**) The mRNA level of *rxr-α* in the liver. (**D**) The mRNA level of *Rxr-α* in the liver. Data are presented as the mean ± SE of the three repeated samples. ## *p* < 0.05, ### *p* < 0.01, #### *p* < 0.001 compared with the control group. * *p* < 0.05, *** *p* < 0.001 compared with HFD group.

Further, RT-qPCR and ELISA experiments were carried out to explore the alterations in *Ppar-γ* and *Rxr-α*. Analysis of the experimental data revealed that the HFD group had 1-fold higher levels of Ppar-γ and a 25% increase in Rxr-α expression relative to the control group (Figure 8). Conversely, treatment with β-sitosterol caused a significant reduction in the expression of both Ppar-γ and Rxr-α (Figure 8). Notably, β-sitosterol exhibited a significant inhibitory effect, reducing Ppar-γ expression by at least 50% and Rxr-α expression by at least 25% in the zebrafish model (Figure 8).

## 4. Discussion

The application of high-fat feed in aquaculture production has become a common practice as it conserves limited and scarce protein resources for energy purposes [44]. However, the adoption of such a diet may cause metabolic challenges in farmed aquatic organisms, which can potentially affect their overall health [5]. Such a diet may result in the accumulation of liver fat and the development of fatty liver [45], metabolic dysfunctions [46,47], and other complications in aquatic organisms. Research has demonstrated that a high-fat diet can stimulate the oxidation of fatty acids and glucose in species of *Larimichthys crocea*, leading to the accumulation of lipids [48]. Consumption of a high-fat diet has been shown to limit the growth and liver lipid accumulation in *Micropterus salmoides* [49]. Therefore, identifying substances that ameliorate the adverse effects of high-fat diets on aquatic organisms is an important research endeavor in the field of aquaculture. The natural compound β-sitosterol has been shown to reduce blood lipid and cholesterol levels. Therefore, we employed zebrafish as the experimental model to explore the role of β-sitosterol in lipid metabolism regulation, with the aim of providing empirical evidence to guide the utilization of β-sitosterol as an aquatic feed supplement.

Therefore, an NAFLD model of zebrafish larvae was constructed by HFD induction. The efficacy of β-sitosterol in reducing triglycerides and cholesterol was examined through oil red O staining and Filip staining. Observable characteristics of zebrafish were used as markers to indicate changes in triglyceride and cholesterol levels among adult and juvenile fish under varying experimental conditions. Our results show that administering a high-fat diet (8% cholesterol) to adult zebrafish and zebrafish larvae resulted in an excess accumulation of triglycerides and cholesterol within their bodies (Figure 1). This aligns with the outcomes of a high-fat zebrafish model developed by Yan Kong, which involved feeding zebrafish a diet containing 4% cholesterol [50]. After 5 days of consumption of β-sitosterol using the aforementioned model, the accumulation of triglycerides and cholesterol in zebrafish was decreased, which matched with the lipid-lowering properties of the drug bezafibrate (Figure 1). This suggests that β-sitosterol can reduce the levels of triglyceride and cholesterol. In a previous study, β-sitosterol was found to potentially decrease plasma total cholesterol (from 340.3 ± 31 mg/dL to 272.7 ± 41.7 mg/dL) and triglyceride (from 208.8 ± 69.3 mg/dL to 151 ± 46.2 mg/dL) in the hamster model of hypercholesterolemia induced by 0.2% cholesterol diet [51]. Similarly, in a rat model, β-sitosterol caused a significant reduction of blood lipid levels [52]. In goldfish (*Carassius auratus*), intraperitoneal injection of 200 μg/g β-sitosterol resulted in a 50% reduction in cholesterol concentration in the gonads compared to the control group [53]. These results are consistent with the present findings. In the *pdx*1^−/−^ zebrafish mutant model with diabetes, impaired glucose homeostasis caused excessive branching and extension of neovascularization in the retina, accompanied by increased blood vessel diameter [28]. Moreover, in the larval stage of zebrafish, these vascular abnormalities have shown responsiveness to pharmacological interventions aimed at angiogenesis and hyperglycemia [25]. Hence, the change in lens vascular diameter may serve as an assessment metric for evaluating the lipid-reducing effectiveness of pharmaceuticals in zebrafish models with hyperglycemia and hyperlipidemia. Our findings indicate that β-sitosterol can significantly reduce the diameter of blood vessels in the lens of zebrafish (Figure 2A,B), indicating that it can potentially mitigate lipid accumulation in blood vessels and effectively lower blood lipids.

Having confirmed the impact of β-sitosterol on triglyceride and cholesterol levels in juvenile zebrafish models, we further aimed to develop a type 2 diabetes model utilizing the same diet induction method. Next, lipidomics analysis was performed to investigate the effect of β-sitosterol on lipid metabolism in adult zebrafish. To increase the visibility of triglyceride and cholesterol changes in zebrafish, a higher concentration of 200 μg/mL β-sitosterol was utilized in the juvenile zebrafish model. Nevertheless, considering practical production and application costs, a dosage of 0.4 mg/g of β-sitosterol was selected for the adult fish model. In the adult fish model, we quantified the levels of triglyceride and cholesterol in the liver. We found that there was a nearly two-fold increase in the levels of triglyceride and cholesterol in the liver of zebrafish in the HFD group compared to the control group. However, administration of β-sitosterol decreased the levels of triglyceride and cholesterol, suggesting that even at low concentrations, β-sitosterol could exert a lowering effect on these lipids.

The liver plays a crucial role in the regulation of triglyceride and cholesterol metabolism [50,54]. Consequently, we performed a non-targeted lipidomic analysis of the liver tissue from adult fish. Lipidomics analysis carried out on zebrafish revealed that β-sitosterol mainly affected lipid metabolism and signal transduction pathways (Figure 7D,E). Compared to the HFD group, the group treated with β-sitosterol exhibited upregulated and downregulated steroid biosynthesis and steroid hormone biosynthesis in zebrafish. (Figure 7D,E). This phenomenon may be attributed to the effect of β-sitosterol on diverse metabolites, which alters diverse biological functions. Future investigations based on a combination of transcriptomics or proteomics with metabolomics are needed to explore the mechanism of action of β-sitosterol. In this study, the biological functions such as glycerophospholipid metabolism and sphingolipid metabolism were upregulated in the β-sitosterol group (Figure 7D). Glycerophospholipids, which serve as the primary structural components of biofilms, play key roles in the regulation of cell signal transduction and metabolism [55]. Moreover, sphingolipids are crucial components of biofilm structure [56]. Hence, β-sitosterol might improve zebrafish signal transduction function by enhancing glycerophospholipid and sphingolipid metabolism. Compared to the HFD group, the β-sitosterol group showed a decrease in arachidonic acid metabolism and unsaturated fatty acid biosynthesis in lipid metabolism (Figure 7E). Hyperglycemia stimulates oxidative stress, leading to the occurrence of insulin disorders and diabetes [30]. For example, ROS is known to cause mitochondrial damage and induce a significant reduction in insulin secretion. Oxidative stress alters Ca^2+^ homeostasis, thereby increasing Ca^2+^ influx, which triggers the activation of phospholipase to produce arachidonic acid [30]. Furthermore, arachidonic acid, which can be converted into pro-inflammatory metabolites such as hydroxyeicosatetraenoic acid, has been linked to hyperlipidemia and induction of inflammatory responses [55]. In our previous studies, we found that β-sitosterol exerts anti-inflammatory and antioxidant effects [14]. Therefore, β-sitosterol can confer anti-inflammatory effects owing to its ability to inhibit arachidonic acid metabolism. In a study based on *Alismatis rhizome* (AR) [55], it was observed that AR mitigated the upregulation of arachidonic acid metabolism induced by HFD, suppressed the expression of pro-inflammatory factors, and exhibited anti-inflammatory properties, which aligns with the effects of β-sitosterol in this investigation.

Studies have demonstrated that peroxisome proliferator-activated receptors (PPARs) can regulate lipid synthesis and decomposition in cells [57]. Specifically, PPARγ upregulates the expression of key adipogenic genes, thereby enhancing fat synthesis and storage in adipose tissue. It interacts with RXRα to regulate gene transcription [58,59]. The PPARγ/RXRα pathway modulates lipid and glucose metabolism in adipocytes and muscle cells [60]. In this study, we found a significant upregulation of *Ppar-γ* and *Rxr-α* expression in the zebrafish model constructed using HFD. Particularly, there was a two-fold increase in expression levels in the HFD group compared to the control group (Figure 8). These findings, in conjunction with those presented in Figure 3, suggest that activation of *Ppar-γ* and *Rxr-α* may contribute to the increase in fat accumulation and establishment of a zebrafish type 2 diabetes model. Similar observations were reported in a rat model induced by HFD, where PPAR-γ and RXR-α expression was increased in the liver [61,62]. In the zebrafish model induced by HFD, and treated with 10 μM 3,4-dichloroaniline (3,4-DCA), the expression of *pparγ* was upregulated by approximately 50% compared to the control group [63]. Furthermore, administration of bisphenol S (BPS) or overfeeding in zebrafish resulted in the accumulation of visceral fat and up to a two-fold increase in *rxrα* expression [64]. These results are consistent with our present findings. However, in contrast to the HFD group, the β-sitosterol group had a 50% reduction in Ppar-γ expression and a 20% reduction in Rxr-α expression (Figure 8). These observations suggest that β-sitosterol inhibits adipogenesis and fat accumulation by suppressing *Ppar-γ* and *Rxr-α* expression, thereby reducing lipid and cholesterol levels. In mice, HFD resulted in the upregulation of *PPAR-γ* expression [65,66]. Conversely, treatment with rose fruit extract inhibited the expression of *PPAR-γ* (by 38.8% compared to the HFD group), thereby preventing lipid accumulation in mice fed on HFD [66]. These findings are similar to those obtained in this study. Given the intricate pathogenic mechanism of elevated glucose and fat levels, several factors may contribute to the aberrant lipid metabolism. Therefore, it is not sufficient to explore genes associated with lipid metabolism while investigating the pathogenic mechanism. With the maturation and development of molecular biology techniques, there is a growing potential to leverage transgenic zebrafish and gene knockout techniques for more comprehensive research. The increase in glucose and lipid levels is driven by other factors beyond aberrant lipid metabolism. Thus, a comprehensive understanding of the pathological mechanism is required to identify other factors involved. The ongoing advancement and refinement of molecular biology techniques provide the potential to further investigate the utility of transgenic zebrafish or other technologies.

## 5. Conclusions

A model of type 2 diabetes was established in zebrafish using a high-sugar and high-fat diet. The efficacy of β-sitosterol in reducing the accumulation of triglycerides and cholesterol in zebrafish, as well as in mitigating the related phenotypic changes caused by the mentioned diet, was confirmed through oil red O and filipin staining. Using non-targeted lipidomics investigations, the effect of β-sitosterol on lipid metabolism in zebrafish was examined to primarily affect lipid metabolism and signal transduction, with a particular emphasis on steroid biosynthesis and steroid hormone biosynthesis. Moreover, β-sitosterol inhibited adipogenesis and fat storage by suppressing the expression of *Ppar-γ* and *Rxr-α*, thereby mediating its lipid-lowering and cholesterol-lowering properties. This study demonstrated that β-sitosterol altered lipid metabolism in fish, suggesting that it can be utilized as a feed supplement to address the issue of superfluous lipid accumulation in aquatic commodities.

## Figures and Tables

**Table 1:** Primer sequence.

Genes	GenBank Accession NO.	Amplicon Length (bp)	Primer Sequences (5′-3′)	
*pparγ*	NM_131467.1	152	F	CTGCCGCATACACAAGAAGA
			R	TCACGTCACTGGAGAACTCG
*rxra*	U29940	128	F	CTGCCAGATAGACAAACGCCA
			R	CATTATCACTCCTCTCCCGACC
*β-actin*	NM_181601.4	131	F	AGGTCATCACCATTGGCAAT
			R	GATGTCGACGTCACACTTCAT

## Data Availability

The raw data supporting the conclusions of this article will be made available by the authors, without undue reservation.

## Supplementary Materials

The following supporting information can be downloaded at: https://www.mdpi.com/article/10.3390/ani14091289/s1, Table S1: Metabolite detailed classification table; Table S2: Metabolite detailed classification table; Table S3: Metabolite detailed classification table.
